# Impairments in mitochondrial dynamics and morphology in skeletal muscle of breast cancer patients after a single paclitaxel administration

**DOI:** 10.1113/EP093922

**Published:** 2026-07-01

**Authors:** Joris Mallard, Elyse Hucteau, Laura Somme, Hervé Bischoff, Valérie Demais, Pauline Silvestrin, Anne‐Laure Charles, Roland Schott, Xavier Pivot, Thomas J. Hureau, Allan F. Pagano

**Affiliations:** ^1^ Exercise Physiology Laboratory Institut Strauss Strasbourg France; ^2^ Biomedecine Research Center of Strasbourg (CRBS), UR 3072, ‘Mitochondria, Oxidative Stress and Muscle Plasticity’ University of Strasbourg Strasbourg France; ^3^ Faculty of Sport Sciences, European Centre for Education, Research and Innovation in Exercise Physiology (CEERIPE) University of Strasbourg Strasbourg France; ^4^ Plateforme Imagerie In Vitro CNRS UPS‐3156, NeuroPole Strasbourg France

**Keywords:** chemotherapy, mitochondrial dynamics, mitochondrial function, muscle biopsies, muscle homeostasis, skeletal muscle deconditioning

## Abstract

Standard chemotherapy regimens for patients with breast cancer are based on epirubicin–cyclophosphamide (EC) and paclitaxel (TAX) administrations. While it has been shown that first EC administration impairs mitochondrial homeostasis, the isolated effects of TAX have not been studied without previous chemotherapy exposure. We conducted a prospective clinical study including five patients with breast cancer who underwent two vastus lateralis muscle biopsies before and 4 days after the first TAX administration, without any prior chemotherapy exposure. Mitochondrial respiratory capacity, reactive oxygen species production, mitochondrial dynamics and ultrastructure, and apoptosis were assessed using high‐resolution respirometry, western blotting, transmission electron microscopy, and TUNEL assay, respectively. Post‐TAX, the number of intermyofibrillar mitochondria decreased (−18%; *P* = 0.049), while the proportion of damaged mitochondria increased (+34%; *P* = 0.012). Mitochondrial area, perimeter and major/minor axis lengths increased (*P *< 0.05) while intermyofibrillar cristae area decreased (4.29% pre‐TAX vs. 2.60% post‐TAX; *P* = 0.044). Despite these morphological changes, oxidative phosphorylation capacity and respiratory control ratio remained unchanged, whereas complex I‐linked substrate respiration decreased (−29%; *P* = 0.046). MFN2 (−43%; *P* = 0.040) and Fis1 (−46%; *P* = 0.046) protein levels decreased post‐TAX while mitophagy markers were unchanged. Apoptosis was increased, as documented by increased Bax (+58%; *P* = 0.045) and TUNEL‐positive nuclei (+395%; *P* = 0.041). In only 4 days, the first TAX administration induced severe skeletal muscle mitochondrial remodelling in patients with breast cancer, characterized by impaired mitochondrial dynamics that resulted in swollen and damaged organelles. These findings demonstrate that mitochondrial toxicity, classically documented at the end of treatment, occurs acutely and after only one chemotherapy administration.

## Abstract (highlights)

- **What is the central question of this study?**
  Does the first administration of paclitaxel acutely affect skeletal muscle mitochondrial homeostasis in patients with breast cancer without prior chemotherapy exposure?
- **What is the main finding and its importance?**
  A single paclitaxel administration induced rapid mitochondrial remodelling in skeletal muscle within 4 days. These findings demonstrate that mitochondrial toxicity, previously documented at the end of chemotherapy or after epirubicin–cyclophosphamide administration, occurs after only one paclitaxel administration in patients without prior chemotherapy exposure. Given the central role of mitochondria in skeletal muscle function, early implementation of preventive strategies is needed.

## 1 INTRODUCTION

Breast cancer is the most commonly diagnosed cancer in women worldwide, and improvements in survival rates have shifted attention toward the long‐term adverse effects of the treatments. Among treatment options, standard chemotherapy typically consists of sequential administrations of epirubicin–cyclophosphamide (EC) and weekly paclitaxel (TAX). These drugs, although effective in targeting tumour cells, cause multiple adverse events, including a severe skeletal muscle deconditioning (Mallard et al., 2021), which increases the mortality risk (Amitani et al., 2022). More precisely, skeletal muscle alterations are characterized by a potent skeletal muscle atrophy (Guigni et al., 2018; Mallard et al., 2024; Mallard, Hucteau, Bender et al., 2022; Mijwel et al., 2018) combined with a dysregulation of mitochondrial homeostasis (Guigni et al., 2018; Mallard et al., 2024; Mallard, Hucteau, Charles et al., 2022; Mijwel et al., 2018), which likely explains the reduction in exercise capacity (Peel et al., 2014).

The effects of chemotherapy on skeletal muscle mitochondria include both structural and functional alterations (Hiensch et al., 2019). Specifically, mitochondrial content was reduced after chemotherapy completion (Guigni et al., 2018; Mallard, Hucteau, Charles et al., 2022; Mijwel et al., 2018), which is consistent with the lower mitochondrial density observed in patients with breast cancer compared with their healthy counterparts (Guigni et al., 2018). In parallel, mitochondrial respiratory capacity was decreased while reactive oxygen species production increased, reflecting an alteration in mitochondrial function (Kunz et al., 2022; Mallard, Hucteau, Charles et al., 2022). Recently, we dissociated the specific effects of each chemotherapy agent used (i.e., EC and TAX) on mitochondrial homeostasis. By performing muscle biopsies before and 4 days after the first administration of each drug (Mallard et al., 2024), we found that only EC reduced mitochondrial function and content. These findings may be explained by impairments in mitochondrial dynamics resulting in an increased apoptotic process (Mallard et al., 2024). While subsequent TAX administration also increased apoptosis, no further alterations were observed in mitochondrial homeostasis. However, patients of the TAX group had already received EC administrations, which could have reduced the effects of TAX on mitochondria. Importantly, several mitochondrial markers such as citrate synthase or VDAC protein expression had not returned to basal levels pre‐TAX compared to pre‐EC values, suggesting a persistent mitochondrial alteration. This hypothesis is strengthened by a preclinical study showing that TAX administration alone reduced mitochondrial content (Guigni et al., 2018).

In this context, clarifying the isolated effect of TAX on human skeletal muscle mitochondria, that is, without prior EC administrations, is essential to provide both fundamental and clinically relevant insights to develop preventive strategies. We therefore conducted a prospective clinical study in patients with breast cancer.

## 2 METHODS

### 2.1 Participants and study design

Patients with breast cancer from the Institut Strauss were included in this prospective clinical study (NCT06536556). Included patients performed two vastus lateralis muscle biopsies: before (pre) and 4 days after (post) the first TAX administration. All participants provided written informed consent prior to enrolment, and the study was conducted in accordance with the *Declaration of Helsinki* and with ethics approval from the national ethics committee (2023‐A01265‐40). The eligibility criteria included non‐pregnant women of at least 18 years of age with a WHO performance status of 0–2 who were diagnosed with breast cancer and treated with (neo)adjuvant TAX chemotherapy. Patients received a TAX administration at 90 mg/m^2^. Due to HER2‐positive tumour status, they concomitantly received trastuzumab at 8 mg/kg.

### 2.2 Skeletal muscle biopsies

Skeletal muscle biopsies were obtained from the left vastus lateralis muscle using a 5 mm Bergström biopsy needle under sterile conditions and local anaesthesia (1% lidocaine). Tissue for mitochondrial respiration, H_2_O_2_ and superoxide anion production measurements was immediately immersed in Krebs–HEPES buffer. Tissue for western blotting analysis was immediately frozen in liquid nitrogen and stored at −80°C. Tissue for histological analyses was embedded in small silicone casts filled with a cryoprotectant agent (OCT compound, Sakura, Finetek, Torrance, CA, USA), immediately cooled in 2‐methylbutane, immersed in liquid nitrogen and stored at −80°C. Tissue for transmission electron microscopy was immediately fixed in 2.5% glutaraldehyde in phosphate buffer.

### 2.3 Mitochondrial respiratory capacity recording

Mitochondrial respiratory capacity recording was performed as previously described (Mallard et al., 2024). Data analysis was performed in duplicate using Oxygraph‐2k‐DatLab software version 6.2, and the results are expressed in pmol/s/mg wet weight.

### 2.4 Reactive oxygen species measurements

Simultaneously with mitochondrial respiratory capacity recording on high‐resolution oxygraphy, H_2_O_2_ production was measured as previously described (Mallard, Hucteau, Charles et al., 2022) with 20 µM Amplex Red and 1 U/mL horseradish peroxidase. Fluorescence was measured in duplicate, and the results are expressed in pmol/s/mg wet weight.

The production of superoxide anion was measured with electron paramagnetic resonance using the specific spin probe 1‐hydroxy‐3‐methoxycarbonyl‐2,2,5,5‐tetramethylpyrrolidine hydrochloride (200 µM), as fully described previously (Mallard, Hucteau, Charles et al., 2022). Superoxide anion production analysis was performed in duplicate using Bruker BioSpin WinEPR Spectrometer software version 4.5 (Bruker BioSpin, Billerica, MA, USA), and the results are expressed in µmol/min/mg dry weight.

### 2.5 Western blotting and antibodies

The western blotting procedures were fully described previously (Mallard, Hucteau, Charles et al., 2022). The following primary antibodies were used: anti‐Bax (Santa Cruz Biotechnology, Dallas, TX, USA, cat. no. Sc‐7480, 1:50), anti‐Bcl‐2 (Santa Cruz, cat. no. Sc‐7382, 1:200), anti‐p‐DRP1 (Ser^616^, Cell Signaling Technology, Danvers, MA, USA, cat. no. 34555, 1:300), anti‐DRP1 (Cell Signaling, cat. no. 5391S, 1:500), anti‐Fis1 (Santa Cruz, Sc‐376447, 1:200), anti‐MFN2 (Santa Cruz, cat. no. Sc‐100560, 1:200), anti‐OPA1 (Thermo Fisher Scientific, Waltham, MA, USA, cat. no. MA5‐16149, 1:1000), anti‐Parkin (Abcam, cat. no. AB77924, 1:500), anti‐PINK1 (Thermo Fisher Scientific, PA1‐16604, 1:500) and anti‐PGC‐1α1 (Millipore, Billerica, MA, USA, cat. no. AB3242, 1:1000). Then, anti‐rabbit (Cell Signaling, 1:4000 cat. no. 7074S) or anti‐mouse (Cell Signaling, 1:4000 cat. no. 7076S) secondary antibodies were used. The blots were revealed using a Pierce ECL kit (Thermo Fisher Scientific) or SupraSignal Femto kit (Thermo Fisher Scientific), and proteins were visualized by enhanced chemiluminescence (iBright 1500 Imaging System, Thermo Fisher Scientific) and quantified with ImageJ Software (version 1.8.0). Ponceau coloration was used as the loading control.

### 2.6 Transmission electron microscopy

Tissues for transmission electron microscopy were post‐fixed in 1% osmium tetroxide in distilled water for 1 h and rinsed with water for 10 min. Samples were then dehydrated through a graded ethanol series and transferred to propylene oxide, the resin solvent, for two successive 10‐min baths. They were subsequently incubated overnight in a 1:1 mixture of propylene oxide and EMBED 812 resin (Electron Microscopy Sciences, Hatfield, PA, USA). After two successive 4‐h baths in pure resin, tissues were placed in moulds filled with resin and polymerized for 30 min at 45°C, then for 2 days at 60°C. The resulting resin blocks were sectioned using an ultramicrotome, and ultrathin sections (50 nm) were contrasted with uranyl acetate and lead citrate before observation with a Hitachi H7500 transmission electron microscope (Hitachi HighTechnologies, Tokyo, Japan)‐ equipped with a Hamamatsu camera (Hamamatsu Photonics, Hamamatsu City, Japan).

Subsarcolemmal and intermyofibrillar mitochondria were investigated using MATLAB 2025b script (MathWorks, Natick, MA, USA). Mitochondria classified as damaged exhibited at least one of the following criteria: discontinuity in the membrane, diffuse cristae, circular shape without clearly identifiable cristae or paracrystalline inclusions (i.e., white deposits). Images were acquired from multiple randomly selected regions of muscle sections and analyses were performed on approximately 200 images per patient and per condition (pre‐ and post‐TAX).

### 2.7 Cryosectioning and histological analyses

Transverse serial cross sections (7 µm) were obtained using a cryostat maintained at −20°C. Then, an in situ cell death detection kit (Merck, Darmstadt, Germany, cat. no. 11684795910) based on TUNEL method was used to quantify single‐cell apoptosis according to the manufacturer's instructions. For each section, all TUNEL‐positive signals were manually quantified and 4′,6‐diamidino‐2‐phenylindole (DAPI) was used to ensure that the TUNEL‐positive signals were co‐localized with a nucleus, while non‐specific signals (e.g., free‐floating or extra‐tissue signals) were not included in the quantification. Results are expressed as an absolute value of TUNEL‐positive nuclei per muscle section. All slides were digitalized with a Zeiss Apotome.2 microscope (Carl Zeiss Microscopy, Oberkochen, Germany) with a ×20 objective (Hamamatsu).

### 2.8 Statistical analysis

Five patients were enrolled in this study. The small sample size reflects the rarity of patients receiving this treatment in the context of HER2‐positive tumour, which limited patient recruitment during the predefined study period (16 months). A posteriori power calculation was performed on the primary outcome (i.e., number of intermyofibrillar mitochondria), which decreased post‐TAX. Based on the observed effect, the estimated power was 0.95, indicating a high probability of detecting a true effect.

The Shapiro–Wilk test was used to check the data normality. A two‐tailed, paired Student's *t*‐test or Wilcoxon's test was then performed to compare pre‐TAX and post‐TAX variables. Statistical significance was set at *P *< 0.05, and all values are expressed as the mean ± standard deviation. Statistical analyses and graphs were made with the GraphPad Prism 10 software (GraphPad Software, Boston, MA, USA).

## 3 RESULTS

### 3.1 Patient characteristics

Five patients were included (59 ± 5 years, 79 ± 19 kg, 29 ± 6 kg/m^2^) with luminal (A or B) and HER2‐positive tumours.

### 3.2 Mitochondrial function

**FIGURE 1:**
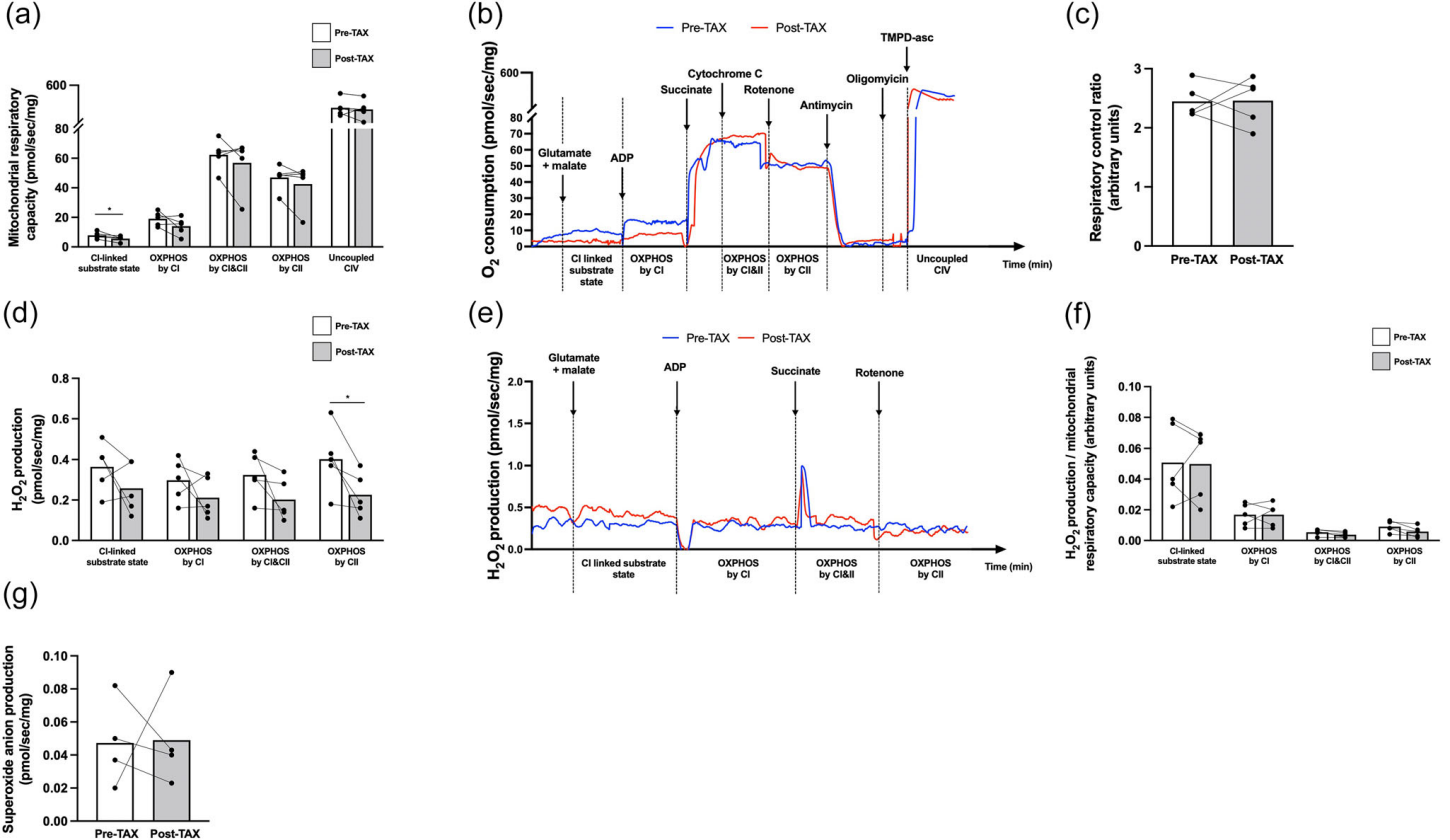
Changes in mitochondrial function. (a, b) Mitochondrial respiratory capacity (a) and representative curves of one patient (b) for CI‐linked substrate state, OXPHOS by CI, OXPHOS by CI and CII, and OXPHOS by CII and uncoupled CIV on permeabilized fibres from vastus lateralis muscle biopsies taken before (pre) and 4 days after (post) the first paclitaxel (TAX) administration. (c–e) Respiratory control ratio (c) and H_2_O_2_ production quantification (d) with representative curves of one patient (e) for the CI‐linked substrate state, OXPHOS by CI, OXPHOS by CI and CII, and OXPHOS by CII in permeabilized fibres. (f) H_2_O_2_ production/mitochondrial respiratory capacity ratio. (g) Superoxide anion measurement through electron paramagnetic resonance in minced muscle samples. For every index, bars correspond to mean group data, and dots correspond to individual data. Five patients were assessed except for superoxide anion production, where 1 patient was excluded because of technical issues during the experimentation. *P < 0.05. CI, complex I; CII, complex II; CIV, complex IV; OXPHOS, oxidative phosphorylation.

Despite a reduction in mitochondrial respiratory capacity in complex I (CI)‐linked substrate state post‐TAX (−29%; *P* = 0.046; Figure 1a, b), oxidative phosphorylation (OXPHOS) was not impaired post‐TAX for OXPHOS by CI (*P* = 0.152), by CI and complex II (CII) (*P* = 0.360) or by CII (*P* = 0.438) and for uncoupled by complex IV (CIV) (*P* = 0.565). Similarly, the respiratory control ratio remained unchanged (*P* = 0.956; Figure 1c).

H_2_O_2_ production did not change post‐TAX for all substrate additions, except for OXPHOS by CII where production was decreased (−44%; *P* = 0.036; Figure 1d, e). However, when normalized to mitochondrial respiratory capacity (Figure 1f), H_2_O_2_ production was unchanged post‐TAX. Similarly, anion superoxide production did not change post‐TAX (*P* = 0.946; Figure 1g).

### 3.3 Mitochondrial dynamics

**FIGURE 2:**
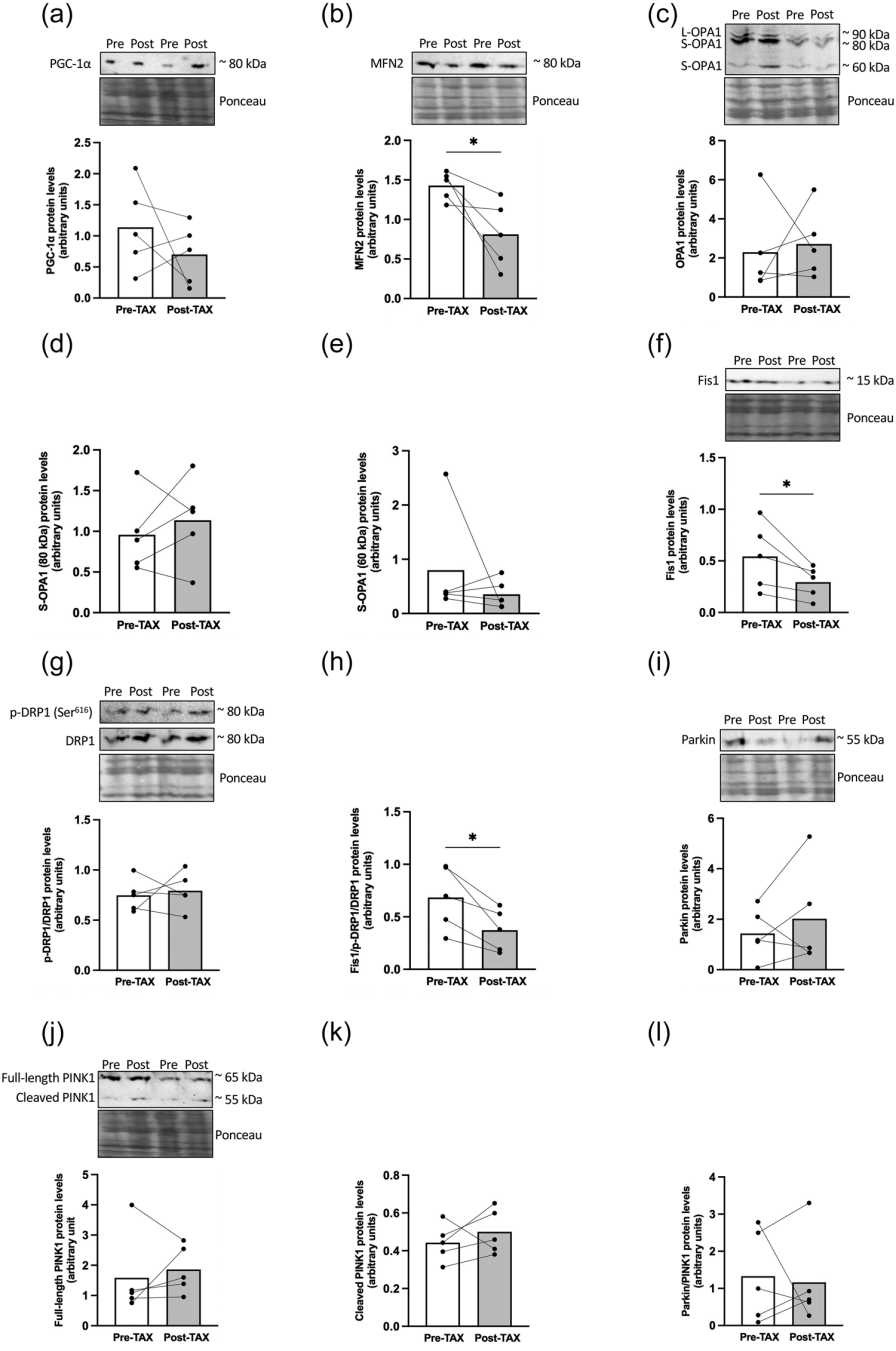
Changes in markers of mitochondrial dynamics and mitophagy. (a–g) Protein levels of PGC‐1α (a), MFN2 (b), L‐OPA1 (c), S‐OPA1 (80 kDa) (d), S‐OPA1 (60 kDa) (e), Fis1 (f) and p‐DRP1 (Ser^616^)/DRP1 protein levels (g) from vastus lateralis muscle biopsies taken before (pre) and 4 days after (post) the first paclitaxel (TAX) administration. (h) Ratio between Fis1 and p‐DRP1/DRP1 protein levels. (i–l) Protein levels of Parkin (i), full‐length PINK1 (j), cleaved PINK1 (k) and Parkin/full‐length PINK1 ratio (l). Two representative patients are displayed above each panel with western blot analyses. For every index, bars correspond to mean group data, and dots correspond to individual data. Five patients were assessed. *P < 0.05.

PGC‐1α1 protein levels, a co‐transcriptional activator of mitochondrial biogenesis, did not change post‐TAX (*P* = 0.365; Figure 2a). A decrease in MFN2 protein levels was observed (−43%; *P* = 0.040; Figure 2b), with no changes in long (l)‐OPA1 (*P* = 0.438; Figure 2c) and the two short (S)‐OPA1 isoforms (80 kDa; *P* = 0.478; Figure 2d; 60 kDa; *P* = 0.813; Figure 2e). While Fis1 protein levels decreased post‐TAX (−46%; *P* = 0.046; Figure 2f), p‐DRP1^Ser616^/DRP1 protein levels did not change (*P* = 0.725; Figure 2g). Consequently, the Fis1/p‐DRP1^Ser616^/DRP1 ratio decreased (−45%; *P* = 0.020; Figure 2h).

No changes were found in Parkin (*P* = 0.446; Figure 2i) and PINK1 protein levels, either for the full‐length form (*P* = 0.592; Figure 2j) or the cleaved form (*P* = 0.415; Figure 2k). Accordingly, the Parkin/PINK1 ratio was unchanged (*P* = 0.813; Figure 2l).

### 3.4 Mitochondria number and morphology

**FIGURE 3:**
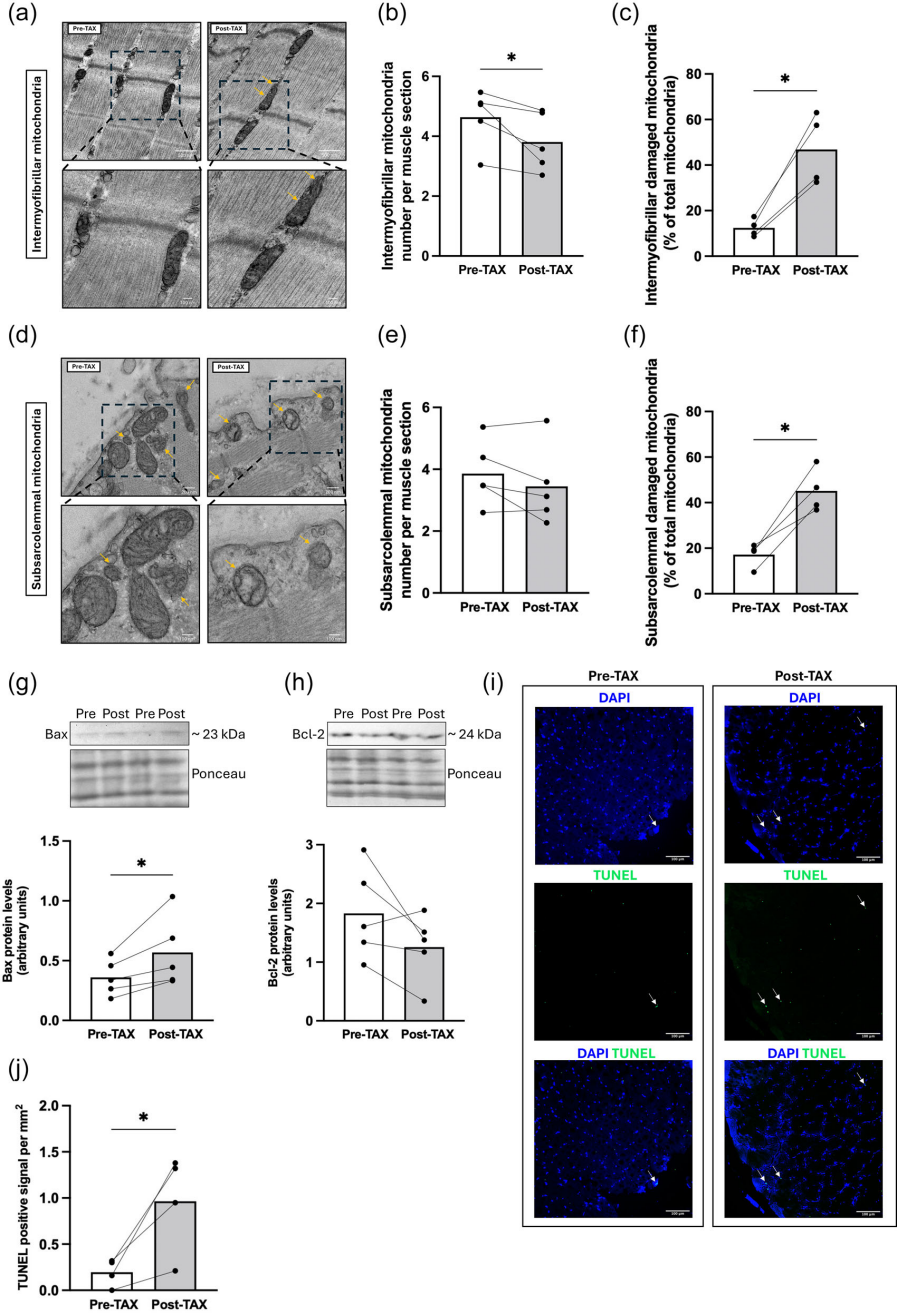
Changes in mitochondrial content and apoptotic process. (a–c) Intermyofibrillar transmission electron microscopy representative images (a), changes in intermyofibrillar mitochondria number (b), and percentage of damaged mitochondria (c). (d–f) Subsarcolemmal transmission electron microscopy representative images (d), changes in subsarcolemmal mitochondria number (e), and percentage of damaged mitochondria (f). Yellow arrows indicate signs of damaged mitochondria, defined as follows: in intermyofibrillar regions, mitochondria showing membrane discontinuity; in subsarcolemmal regions, mitochondria showing membrane discontinuity together with a circular shape without clearly identifiable cristae and/or diffuse cristae. (g–j) Protein levels of Bax (g), Bcl‐2 (h), and TUNEL‐positive signal per mm^2^ section (i, j) with representative transverse muscle sections with DAPI and/or TUNEL assay. For every index, bars correspond to mean group data, and dots correspond to individual data. Five patients were assessed except for subsarcolemmal damaged mitochondria for TUNEL assay where one patient was excluded due to poor image quality. *P < 0.05.

Number of intermyofibrillar mitochondria decreased post‐TAX (−18%; *P* = 0.049; Figure 3a, b) while the percentage of damaged mitochondria increased (+34%; *P* = 0.012; Figure 3c). Despite no changes in the number of subsarcolemmal mitochondria (*P* = 0.194; Figure 3d, e), the proportion of damaged mitochondria also increased (+28%; *P* = 0.011; Figure 3f).

As detailed in Table 1, TAX altered the morphology of both intermyofibrillar and subsarcolemmal mitochondria. However, no changes were found for mitochondrial ultrastructure (Table 1), except for a decrease in the proportion of cristae area relative to total mitochondrial area (4.29% pre‐TAX vs. 2.60% post‐TAX; *P* = 0.044) in intermyofibrillar mitochondria.

**TABLE 1:** Changes in mitochondria shape and ultrastructure.

	Pre‐TAX	Post‐TAX	*P*
**Shape (intermyofibrillar)**			
Area (µm^2^)	0.08 ± 0.02	0.13 ± 0.05	**0.015**
Mitochondrial density (%)	1.01 ± 0.45	1.44 ± 0.36	**0.020**
Perimeter (µm)	1.07 ± 0.19	1.51 ± 0.37	**0.015**
Major axis length (µm)	0.43 ± 0.09	0.64 ± 0.19	**0.019**
Minor axis length (µm)	0.22 ± 0.02	0.26 ± 0.03	**< 0.001**
Circularity (arbitrary units)	0.83 ± 0.07	0.74 ± 0.07	**0.040**
Roundness (arbitrary units)	0.60 ± 0.07	0.50 ± 0.09	0.058
**Shape (subsarcolemmal)**			
Area (µm^2^)	0.11 ± 0.03	0.19 ± 0.04	**0.042**
Mitochondrial density (%)	1.35 ± 0.35	2.12 ± 0.52	**0.024**
Perimeter (µm)	1.30 ± 0.19	1.78 ± 0.26	**0.022**
Major axis length (µm)	0.52 ± 0.08	0.71 ± 0.13	**0.024**
Minor axis length (µm)	0.25 ± 0.03	0.34 ± 0.04	**0.041**
Circularity (arbitrary units)	0.76 ± 0.02	0.75 ± 0.05	0.529
Roundness (arbitrary units)	0.54 ± 0.03	0.54 ± 0.06	0.655
**Ultrastructure (intermyofibrillar)**			
Membrane thickness (nm)	17.97 ± 3.77	18.43 ± 3.93	0.809
Number of cristae per mitochondria	2.13 ± 0.78	2.75 ± 0.97	0.377
Cristae area/mitochondria area (%)	4.29 ± 0.80	2.60 ± 1.02	**0.044**
Cristae major axis length (nm)	119.69 ± 16.02	123.08 ± 17.96	0.557
Cristae minor axis length (nm)	31.55 ± 6.07	28.81 ± 3.96	0.266
Distance between reticulum and mitochondria (nm)	36.29 ± 15.54	45.44 ± 16.79	0.541
**Ultrastructure (subsarcolemmal)**			
Membrane thickness (nm)	16.63 ± 2.54	16.65 ± 1.48	0.973
Number of cristae per mitochondria	4.36 ± 0.67	5.06 ± 0.99	0.184
Cristae area/mitochondria area (%)	1.92 ± 0.26	1.44 ± 0.42	0.157
Cristae major axis length (nm)	138.22 ± 13.27	146.87 ± 25.12	0.539
Cristae minor axis length (nm)	28.84 ± 5.02	31.90 ± 6.01	0.524

### 3.5 Apoptosis

Bax protein levels increased (+58%; *P* = 0.047; Figure 3g), combined with no changes in Bcl‐2 protein levels (*P* = 0.134; Figure 3h), its anti‐apoptotic counterpart. At the histological level, we found an increase in TUNEL‐positive signal (+395%; *P* = 0.041; Figure 3i, j) post‐TAX.

## 4 DISCUSSION

Although TAX is usually administered after EC, HER2‐positive tumour status represents a specific clinical setting in which TAX‐first regimens can be used. Taking advantage of this context, we examined the isolated impact of a single TAX administration on skeletal muscle mitochondria. Here, we documented that TAX disrupted mitochondrial dynamics within 4 days, as documented by reduced fusion and fission markers. Consequently, mitochondrial morphology and ultrastructure were altered, resulting in a higher proportion of damaged mitochondria and a reduction in overall mitochondria number.

### 4.1 TAX administration reduced the number of mitochondria without affecting their function

Within only 4 days, TAX administration reduced mitochondria number, particularly those located in the intermyofibrillar compartment. These results contrast with previous findings reporting that patients with breast cancer exhibited a decrease in subsarcolemmal mitochondria but no changes in intermyofibrillar mitochondria at the end of chemotherapy treatment compared to healthy participants (Guigni et al., 2018). This discrepancy may be explained by differences in study design. Patients in Guigni et al. (2018) were compared to controls after chronic treatment involving multiple chemotherapy agents, whereas we conducted longitudinal and acute measurements after a single TAX administration. This differential susceptibility to TAX exposure between intermyofibrillar and subsarcolemmal mitochondria in patients treated with TAX may be related to the higher energetic demand and metabolic load of intermyofibrillar mitochondria (Ferreira et al., 2010), making them more vulnerable to chemotherapy‐induced mitochondrial dysfunction. Indeed, these two subpopulations of mitochondria have distinct physiological roles (Ferreira et al., 2010). Intermyofibrillar mitochondria are more implicated in oxidative phosphorylation and ATP production for contractile activity, whereas subsarcolemmal mitochondria are positioned near the cell periphery to play a greater role in substrate exchange. Of note, recent work further refined the characterization of subsarcolemmal mitochondria by identifying a perivascular mitochondrial (PVM) subpopulation located in close proximity to capillaries and exhibiting distinct morphological and functional properties compared with other subsarcolemmal mitochondria (Parry et al., 2024). However, the identification of PVM relies on 3D imaging approaches allowing precise spatial localization relative to capillaries, whereas our analyses were performed using conventional 2D transmission electron microscopy sections. Therefore, we cannot exclude that this mitochondrial subpopulation may have been differentially affected by TAX.

While mitochondrial content did not further decrease following TAX in patients who had previously received EC (Mallard et al., 2024), the present study documented that TAX can still have a deleterious consequence on the mitochondria when administered first. This suggests that the first exposure to chemotherapy is particularly harmful, rather than the administered agent specificity, in reducing skeletal muscle mitochondrial content in patients with breast cancer. Given the correlation between mitochondrial volume and maximal exercise capacity (Weibel et al., 2004), decreased mitochondrial content or mitochondria number likely explains the decrease in exercise capacity classically observed in patients with breast cancer (Bolam & Howden, 2025; Mallard et al., 2023; Peel et al., 2014). Moreover, with fewer mitochondria available, each organelle has to sustain a higher workload for a given exercise intensity, which may increase the accumulation of metabolites, a key component of neuromuscular fatigue (Hureau et al., 2022). Therefore, the decrease in mitochondrial content likely explains, at least in part, the increase in neuromuscular fatigue in patients with cancer and the increase in perceived fatigue (Brownstein et al., 2022; Mallard et al., 2025).

The reduction in number of intermyofibrillar mitochondria, which support energy production for contractile activity, accounted for the decrease in mitochondrial respiratory capacity in CI‐linked substrate state. However, OXPHOS capacity was not statistically reduced post‐TAX, suggesting that the remaining mitochondria compensated to maintain global OXPHOS efficiency. This interpretation is supported by the steady respiratory control ratio observed post‐TAX, indicating preserved OXPHOS efficiency relative to electron transport despite the reduction in mitochondrial content. Of note, respiratory control ratio values in the present study were similar to those previously reported in patients with inflammatory myopathies (Meyer et al., 2017), suggesting a pre‐existing alteration. Given the relationship between mitochondrial function and exercise capacity, these findings are consistent with the reduced exercise capacity previously reported in patients with breast cancer prior to treatment initiation (Peel et al., 2014). In parallel, H_2_O_2_ production decreased proportionally to respiration, as reflected by the unchanged H_2_O_2_/respiration ratio despite a decrease in absolute H_2_O_2_ production. Therefore, the reduction in absolute H_2_O_2_ production primarily reflects the loss of number of mitochondria rather than an intrinsic change in ROS‐generating capacity per mitochondrion, as further supported by stable anion superoxide production, the primary mitochondrial ROS precursor of H_2_O_2_. When preceded by EC administrations, TAX increased H_2_O_2_ production without affecting mitochondrial content or respiratory capacity (Mallard et al., 2024), indicating that chronic chemotherapy exposure, rather than acute administration, is the main driver of increased ROS production. This interpretation is supported by a previous study reporting elevated H_2_O_2_ levels at the end of chemotherapy treatment in patients with breast cancer (Mallard, Hucteau, Charles et al., 2022).

### 4.2 TAX administration impaired mitochondrial dynamics

Despite no changes in mitochondrial biogenesis, we found a decrease in fusion and fission markers, indicating impairments in mitochondrial dynamics. Under physiological conditions, mitochondrial homeostasis relies on the coordination of biogenesis, fusion, fission and mitophagy. Newly synthetized mitochondria can fuse with pre‐existing ones, leading to larger organelles. During fission, damaged regions of mitochondria are separated and subsequently degraded through mitophagy, thereby maintaining a healthy mitochondrial network. In this study, we found a decrease in MFN2 protein levels, indicating a plausible dysregulation in the outer‐membrane fusion process. Although OPA1 protein levels remained unchanged for both long and short isoforms, suggesting that inner membranes fusion was preserved, the coordination between outer and inner membranes fusion is needed for healthy mitochondria (Pernas & Scorrano, 2016). Therefore, our results suggest an overall impairment of mitochondrial fusion following TAX treatment, an alteration already observed after the first EC administration (Mallard et al., 2024). These results indicate that, regardless of the type of chemotherapeutic agent used, the first administration is sufficient to impair the fusion process. We also documented that mitochondrial fission regulators were differentially modulated by TAX: Fis1, involved in DRP1 recruitment, decreased post‐TAX, whereas p‐DRP1^Ser616^/DRP1, reflecting DRP1 activation, remained unchanged. These findings suggest an attenuation of mitochondrial fission signalling, supporting by the decrease in Fis1/DRP1^Ser616^/DRP1 ratio, potentially limiting the recruitment of the fission machinery for proper mitochondrial remodelling. Given that fission is a prerequisite for mitophagy (Kubli & Gustafsson, 2012), the limitation of the fission process would typically be expected to impair mitophagy processes. Despite altered mitochondrial dynamics‐related proteins, PINK1 and Parkin protein levels remained unchanged following TAX administration. However, we observed a higher proportion of damaged mitochondria, suggesting that preserved PINK1/Parkin protein expression was not sufficient to maintain mitochondrial quality control. Together, these findings indicate that TAX administration impairs mitochondrial dynamics, as evidenced by the reduced fusion and fission signalling, potentially disrupting the ability of the mitochondrial network to adapt to cellular stress. This reduced plasticity likely promotes the accumulation and persistence of damaged mitochondria within skeletal muscle.

### 4.3 Impairments in mitochondrial dynamics modulate mitochondrial morphology and apoptosis

Alterations in mitochondrial dynamics have been associated with impaired mitochondrial quality control and the accumulation of damaged mitochondria in various pathological contexts (Eisner et al., 2018; Twig et al., 2008; Yapa et al., 2021), which is consistent with our observations indicating a higher proportion of damaged mitochondria post‐TAX. In addition, given the impaired mitochondrial dynamics observed after TAX, changes in mitochondrial morphology were also expected (Leduc‐Gaudet et al., 2021). Classically, reduced mitochondrial fusion is associated with mitochondrial fragmentation, while impaired fission leads to the accumulation of elongated mitochondria (Leduc‐Gaudet et al., 2021). However, in the present study, we observed a concomitant reduction in both fusion‐ and fission‐related markers. Rather than suggesting a neutral effect on mitochondrial morphology, such a pattern is more consistent with an overall reduction in mitochondrial network plasticity and turnover. Indeed, mitochondrial morphology is not only determined by the direction of fusion versus fission imbalance, but rather by the interaction between these factors and the overall efficiency of mitochondrial dynamics (Twig et al., 2008). We observed an increase in mitochondrial area, together with higher perimeter and major/minor axis lengths. Thus, the increased mitochondrial size observed here should not be interpreted as a marker of improved mitochondrial health or adaptive remodelling, but rather as persistence and accumulation of damaged and structurally altered mitochondria. This interpretation is further supported by our results found through transmission electron microscopy analysis, which documented an increase in the percentage of damaged and swollen mitochondria post‐TAX.

Mitochondrial swelling is a hallmark of mitochondrial dysfunction‐associated apoptosis (Wakabayashi, 1999), which was further strengthened in this study by a reduction in the proportion of cristae area and an increase in damaged and diffuse cristae. These structural alterations are typically associated with the pro‐apoptotic activity of Bax (Vringer & Tait, 2023), whose protein expression was increased after TAX administration without changes in Bcl‐2, its anti‐apoptotic counterpart. Ongoing apoptosis was further supported by the increase in TUNEL‐positive nuclei at the histological level. However, it should be considered that skeletal muscle tissue contains several non‐muscle cell populations, and our experiments do not allow discrimination between myonuclei and nuclei from other resident cell types within the muscle environment. That said, we previously documented that TAX administration, preceded by EC exposure, was associated with an increase in TUNEL staining in skeletal muscle, likely in response to H_2_O_2_ production (Mallard et al., 2024). Here, we reported that H_2_O_2_ levels were not increased, indicating that apoptosis may have been initiated by alternative mechanisms. Among these, structural ruptures in mitochondrial membranes are known to trigger the release of cytochrome *c* into the cytosol, which in turn promotes apoptosome formation, thereby committing the cell to apoptosis. Accordingly, mitochondria exhibiting ruptures in their membranes were classified as damaged, and we documented an increased proportion of such mitochondria post‐TAX. In addition, cytochrome *c* release into the cytosol may also be regulated by the mitochondrial permeability transition pore, a process that can also promote apoptosis (Sesso et al., 2004) and may constitute an alternative mechanism involved in TAX‐induced apoptosis. Therefore, skeletal muscle apoptosis appears as a hallmark of TAX administration that might be triggered through distinct mechanisms whether TAX is given first or after EC in patients with breast cancer.

Increased apoptosis in skeletal muscle likely explains the decrease in number of mitochondria observed 4 days after TAX. However, it should be noted that a study conducted in a preclinical model of skeletal muscle deconditioning documented a decrease in mitochondrial biogenesis preceding the reduction in mitochondrial content (Leermakers et al., 2019). Thus, as mitochondrial biogenesis is a highly dynamic process that can be regulated within hours following a stimulus, we cannot exclude that a reduction in mitochondrial biogenesis was missed by performing the second skeletal muscle biopsy 4 days after TAX administration.

### 4.4 Methodological considerations

This study documented that a single TAX administration is sufficient to induce severe mitochondrial alterations in the skeletal muscle of patients with breast cancer within only 4 days. Despite the small sample size (*n* = 5), due to the rarity of patients initiating treatment with TAX and the short inclusion period, power calculation for the primary outcome (i.e., intermyofibrillar mitochondrial number) suggested a high probability of detecting an effect (0.95). In addition, the pre–post design effectively minimized interindividual variability and all analyses revealed convergent alterations across multiple independent measures. It should also be noted that patients with HER2‐positive tumours concomitantly received trastuzumab, a monoclonal antibody targeting HER2 receptors. Among muscle tissues, only cardiac muscle appears to be affected by trastuzumab because it abundantly expresses HER2 receptors (Nicolazzi et al., 2018). However, evidence supporting the effects or the absence of effects of trastuzumab on skeletal muscle remains very limited. Therefore, a specific contribution of trastuzumab, or potential combined treatment effects with TAX, on skeletal muscle mitochondria cannot be formally excluded and further studies are needed to decipher the specific role of trastuzumab.

### 4.5 Clinical applications

Given the central role of mitochondria in health, preventive strategies should be implemented prior to the initiation of chemotherapy. Aerobic exercise is currently the most effective strategy to enhance mitochondrial function by promoting mitochondrial biogenesis (Little et al., 2011) and improving mitochondrial morphology (Picard et al., 2013). In patients with breast cancer, only one study documented that exercise increased citrate synthase activity (Mijwel et al., 2018), an indirect marker of mitochondrial content, and preserved ${\dot{V}}_{O_{2}\max}$, whereas the control group exhibited declines.

### 4.6 Conclusion

In only 4 days, a single TAX administration induced severe skeletal muscle mitochondrial remodelling in patients with breast cancer, characterized by impaired mitochondrial dynamics that resulted in swollen and damaged organelles. These findings demonstrate that mitochondrial toxicity documented at the end of treatment occurs after only one chemotherapy administration. Given the central role of mitochondria in muscle function, implementing preventive strategies may be essential to preserve patients’ functional capacity.

## AUTHOR CONTRIBUTIONS

Joris Mallard and Allan F. Pagano conceived the study. Joris Mallard, Allan F. Pagano, Elyse Hucteau, Laura Somme and Hervé Bischoff performed the skeletal muscle biopsies. Joris Mallard and Allan F. Pagano performed the histological analyses. Pauline Silvestrin and Anne‐Laure Charles, performed mitochondrial function experiments. Valérie Demais performed the transmission electron microscopy experiments and Elyse Hucteau analyzed the images. Joris Mallard and Allan F. Pagano performed western blot analyses. Joris Mallard, Elyse Hucteau, Allan F. Pagano and Thomas J. Hureau interpreted the results. Joris Mallard, Elyse Hucteau and Allan F. Pagano supervised the study. Joris Mallard, Elyse Hucteau, Thomas J. Hureau, Allan F. Pagano, Roland Schott and Xavier Pivot managed project administration and funding acquisition. Joris Mallard and Allan F. Pagano wrote the manuscript. All the authors approved the results and revised the manuscript. All authors have read and approved the final version of this manuscript and agree to be accountable for all aspects of the work in ensuring that questions related to the accuracy or integrity of any part of the work are appropriately investigated and resolved. All persons designated as authors qualify for authorship, and all those who qualify for authorship are listed.

## CONFLICT OF INTEREST

The authors declare that they have no competing interests.

## Data Availability

De‐identified data are available from the corresponding authors upon reasonable request. In a case of approval, a specific agreement between the sponsor (Institut Strauss) and the researcher may be required for data transfer.
